# EEG-Based Automatic Classification of ‘Awake’ versus ‘Anesthetized’ State in General Anesthesia Using Granger Causality

**DOI:** 10.1371/journal.pone.0033869

**Published:** 2012-03-22

**Authors:** Nicoletta Nicolaou, Saverios Hourris, Pandelitsa Alexandrou, Julius Georgiou

**Affiliations:** 1 Department of Electrical and Computer Engineering, KIOS Research Centre, University of Cyprus, Nicosia, Cyprus; 2 Department of Electrical and Computer Engineering, Holistic Electronics Research Laboratory, University of Cyprus, Nicosia, Cyprus; 3 Nicosia General Hospital, Nicosia, Cyprus; Queensland Brain Institute, Australia

## Abstract

**Background:**

General anesthesia is a reversible state of unconsciousness and depression of reflexes to afferent stimuli induced by administration of a “cocktail” of chemical agents. The multi-component nature of general anesthesia complicates the identification of the precise mechanisms by which anesthetics disrupt consciousness. Devices that monitor the depth of anesthesia are an important aide for the anesthetist. This paper investigates the use of effective connectivity measures from human electrical brain activity as a means of discriminating between ‘awake’ and ‘anesthetized’ state during induction and recovery of consciousness under general anesthesia.

**Methodology/Principal Findings:**

Granger Causality (GC), a linear measure of effective connectivity, is utilized in automated classification of ‘awake’ versus ‘anesthetized’ state using Linear Discriminant Analysis and Support Vector Machines (with linear and non-linear kernel). Based on our investigations, the most characteristic change of GC observed between the two states is the sharp increase of GC from frontal to posterior regions when the subject was anesthetized, and reversal at recovery of consciousness. Features derived from the GC estimates resulted in classification of ‘awake’ and ‘anesthetized’ states in 21 patients with maximum average accuracies of 0.98 and 0.95, during loss and recovery of consciousness respectively. The differences in linear and non-linear classification are not statistically significant, implying that GC features are linearly separable, eliminating the need for a complex and computationally expensive non-linear classifier. In addition, the observed GC patterns are particularly interesting in terms of a physiological interpretation of the disruption of consciousness by anesthetics. Bidirectional interaction or strong unidirectional interaction in the presence of a common input as captured by GC are most likely related to mechanisms of information flow in cortical circuits.

**Conclusions/Significance:**

GC-based features could be utilized effectively in a device for monitoring depth of anesthesia during surgery.

## Introduction

General anesthesia is a drug-induced reversible state of unconsciousness and depression of reflexes to afferent stimuli [1]. The precise mechanisms by which anesthetics disrupt consciousness are difficult to identify. This is partly down to the fact that general anesthesia is a multi-component process, whereby additional desirable components are immobility, analgesia and amnesia. In modern surgery this multi-component process of anesthesia is achieved through the administration of a combination of chemical agents. For example, neuromuscular blocking agents cause muscle paralysis through inhibition of neuronal transmission to muscles. The chemical agents administered have different molecular targets and different effects on the brain. Therefore, this co-administration of such diverse chemical agents, with different methods of action, constitutes the identification of the exact mechanism of anesthesia-induced unconsciousness difficult.

An insight into the complex process of general anesthesia can be obtained through studying how the administration of this chemical “cocktail” affects the observed brain activity. The action of the anesthetic agents causes measurable effects on the brain activity, which can be observed through methods such as the electroencephalogram (EEG). The use of EEG monitors during anesthesia has allowed the identification of some characteristics that are related to the administration of anesthetic agents. For example, anesthesia causes characteristic changes in the spectral content of the EEG: as the depth of anesthesia increases, the faster α (8–12 Hz) and β (12.5–30 Hz) brain rhythms are replaced by slower δ (1.5–3.5 Hz) and θ (3.5–7.5 Hz) activity. In very deep anesthesia the EEG may develop a peculiar pattern of activity known as burst suppression, during which alternating periods of normal to high activity and low voltage (or even isoelectricity) are observed [2]. Despite the usefulness of such observations, they still do not help us understand the physiological mechanisms behind these observed changes, as some of these characteristics are not unique to anesthesia. For this purpose, one must use measures that capture the underlying interactions within the brain as these are manifest in the observed brain activity. One such example is the use of coherence to reveal how the patterns of interaction in the brain are altered during anesthetic-induced loss of consciousness (LOC): it was found that anesthetics disrupt the coherence of neural signals in the γ band [3], [4]. Other similar studies reveal that anesthetics do not block incoming sensory information from reaching the brain, but their administration disrupts the process by which our perceptions are combined into a unified experience (cognitive binding) [4]. Specific brain structures that are integral to this process have been identified through imaging (positron emission tomography – PET) studies using different anesthetic agents [5], [6]. These studies place the thalamus and the neural networks that regulate its activity into a key role in anesthetic-induced unconsciousness, independent of the type of agent utilized.

In addition to studying the mechanisms of general anesthesia and the effects of different anesthetic agents, the use of brain activity has an additional and more direct clinical application: it provides a means of monitoring the depth of anesthesia (DOA) during surgery. The combination of agents and the doses at which these are administered are very much dependent on patient characteristics and surgery requirements, therefore each case is unique. As a result, there are no direct instructions that the anesthetist can follow, but only rough guidelines. Thus, DOA monitors provide an objective method of assessing the state of hypnosis of the patient and provide a useful and welcome aid for the anesthetist. The main concerns of the anesthetist are over- and under-dose of anesthetic agents. Both could have serious implications for the patient. Over long periods over-administration can be costly in terms of agent usage and because of increased patient recovery time. In the worst case, overdosage can lead to death. Underdosage can lead to regaining of consciousness during surgery, which is extremely traumatic. Costs involved with underdosage are related to post-traumatic stress therapy and compensation claims. Intra-operative awareness has been confirmed in a number of cases, with incidence ranging from 0.11–0.8% [7]; however, due to the amnesic effect of certain anesthetics, some patients have no recollection of regaining awareness, therefore it is likely that the actual incidence of awareness is higher than that reported. The incidence of awareness is affected by a number of factors, including the type of surgery, patient characteristics and equipment failure [8], [9]. The use of DOA monitors during surgery could provide a valuable means of identifying awareness during surgery, particularly since the patient himself cannot communicate this to the anesthetist due to immobility from the co-administration of neuromuscular blocking agents.

Currently EEG-based DOA monitors are being introduced for routine patient monitoring during surgery. The most commonly used commercially available devices include the BIS® monitor (Aspect Medical Systems, Natick, MA) [10] and Datex-Ohmeda S/5™ Entropy Module (originally by Datex-Ohmeda Division, Instrumentation Corp., Helsinki; now with GE Healthcare) [11]. These devices operate by converting some specific combination of EEG characteristics into a single number from 0–100 representing the level of hypnosis (with 100 denoting ‘fully awake’ and 0 denoting ‘isoelectricity’). Despite the potential usefulness of such monitors, current technology still suffers from a number of reliability issues. Some monitors are unable to differentiate between the EEG of somebody who is either anesthetized or asleep [12], [13], [14], while others remain unresponsive to specific anesthetic agents [15], [16] or are affected by the administration of other drugs, such as neuromuscular blocking agents [17], [18].

This is due to the fact that the operation of current monitors is based on features that are characteristic of the observed changes in the EEG activity, which may not be a direct reflection of the actual physiological process underlying general anesthesia and which are not unique to anesthetic-induced LOC. However, the measures utilized must be based on ‘neurobiologic phenomena that represent the *necessary* and *sufficient* conditions for consciousness in a specific individual’ [16]. A number of measures that can capture deeper interactions within the brain as these are manifest in the observed brain activity have been developed. More specifically, measures that can reliably capture the changing interactions between different brain areas can provide important insight into how the administration of anesthetic agents affects information flow in the brain. The identification of interruption of cognitive binding as a general mechanism of action of anesthetic agents, independent of the type of agent utilized, implies that measures reflecting this mechanism would result in more reliable and generalized monitors.

In this work Granger Causality (GC), a measure quantifying causal interactions between two time series, is utilized as a feature for discriminating awake from anesthetized state. The main focus of the study was the use of GC as a discrimination feature to capture reversible changes with loss and recovery of consciousness, regardless of the anesthetic protocol used. Our previous investigations showed that GC captures such reversible anesthetic-induced changes in brain activity [19], [20]. These observations support the use of GC as a feature for discriminating between awake and anesthetized state in a DOA monitor.

## Methods

### Dataset

The dataset used in this study was collected from 21 male patients (mean age 37.6±19.1) who underwent routine general surgery at the Nicosia General Hospital, Cyprus. The administration of general anesthesia was not confined to a particular anesthetic regime. The study was approved by the National Bioethics Committee of Cyprus and the patients gave written informed consent for their participation. Participants were not previously taking any medication that influences the central nervous system and were of normal weight. One patient was diagnosed with multiple sclerosis (very early stage). However, the data of this patient were not excluded from the study as the findings were similar with other patients. General anesthesia was induced by the on duty anesthetist using the regular procedures of the hospital. Standard monitoring devices, including pulse oximetry, electrocardiogram, and non-invasive blood pressure, were utilized. All patients were preoxygenated via a face mask prior to anesthesia induction with a Diprivan (propofol 1%, 10 mg/ml) bolus. The induction dose varied from 2 mg/kg to 4 mg/kg depending on patient characteristics. During induction some patients also received boluses of neuromuscular blocking agents (cisatracurium, rocuronium, or atracurium) and analgesic drugs. Depending on patient characteristics and surgery requirements maintenance of anesthesia was achieved with an intravenous administration of propofol at concentrations ranging between 20–50 ml/h (200–500 mg/h). For 2 patients (S12 and S15) maintenance was performed with an inhalational administration of sevoflurane (1–2%). In most patients this was titrated with an intravenous administration of remifentanil hydrochloride (Ultiva®; 2 mg, dissolved in 40 ml) throughout surgery at a rate ranging between 2–15 ml/h (0.1–0.75 mg/h). Following induction of anesthesia the patients' trachea was intubated and surgery commenced. Lungs were ventilated with an air-oxygen or air-oxygen-N_2_O mixture. During surgery boluses of neuromuscular blocking agents and other drugs, such as antibiotics, were administered as required and depending on surgery requirements.

EEG data were collected using the TruScan32 system (Deymed Diagnostic) at a sampling rate of 256 Hz. Electrodes were placed at positions Fp1, Fp2, F7, F3, Fz, F4, F8, T3, C3, Cz, C4, T4, T5, P3, Pz, P4, T6, O1 and O2, according to the International 10/20 system, and were recorded with an FCz reference. No filtering was performed during or after data collection; this is to ensure that the timing relations on which GC depends on are not disrupted by the introduction of causal artifacts from filtering [21]. Data recording usually commenced while patients were still awake prior to administration of the anesthetic agents and continued throughout the entire surgery, until the patient regained consciousness (ROC). For the purpose of this study, the point at which the anesthetic bolus for induction was administered was defined as ‘loss of consciousness’ (LOC); the point at which the patients stopped responding verbally to commands by the anesthetist occurred approximately 10–30 s after administration of the anesthetic bolus, depending on patient characteristics. The main reasoning for specifying LOC in this way is that, firstly, defining the exact point at which patients lose consciousness is a subjective process (as opposed to the point at which the anesthesia bolus is physically administered); and, secondly, loss of consciousness occurs relatively rapidly when induction is performed with a bolus of anesthetic. However, ROC must be defined as the point at which the patient responds to verbal commands or tactile stimuli by the anesthetist, as there is no other precise marker that defines it. Patient response was expressed either as voluntary muscular movement in response to a command by the anesthetist or a verbal response. Throughout the recording, timestamps indicating important events, such as LOC, ROC and bolus agent administration, were manually inserted in the digital EEG record. These markers are necessary for subsequent data analysis and aligning the occurrence of the same events in different EEG recordings.

### Data analysis

The main function of a depth of anesthesia (DOA) monitor is to alert the anesthetist when a subject becomes aware during surgery. Therefore, a minimal requirement for a DOA monitor is the ability to distinguish between the two states ‘Awake’ and ‘Anesthetized’. The ability to classify these two states using GC as a feature was investigated following the methodology described below.

#### EEG segment extraction

Data from 21 subjects were available for analysis (S1–S21). Using the dataset described above, segments of a few minutes duration corresponding to the two classes were extracted from the continuous EEG recordings. Such data is available both at initial loss of consciousness at induction, and recovery of consciousness at the end of surgery. The segments were extracted based on the manual markers inserted in the EEG record during surgery, indicating anesthetic induction and recovery of consciousness. Loss of consciousness after anesthetic induction is patient-dependent and occurs 10–30 s after administration of the anesthetic bolus. In the following analysis we did not use the first 5 minutes of data after the marker for anesthetic induction; this ensured that the data used corresponded to the patient being fully unconscious, and did not contain any artifacts caused from tracheal intubation.

#### Dimensionality reduction

The original data space is 19-dimensional (number of electrodes). In order to reduce this, five brain areas were defined as the average activity of specified electrode grids. The five brain areas defined were: left frontal (LF: electrodes Fp1, F7, F3, T3, C3), right frontal (RF: Fp2, F8, F4, C4, T4), left posterior (LP: T5, P3, O1), right posterior (RP: T6, P4, O2), and midline (Z: Fz, Cz, Pz). The rationale behind these groupings was that fronto-posterior interactions appear to play an important role in (un)consciousness, thus we performed grouping of activity from frontal and posterior areas in order to investigate such fronto-posterior interactions. Electrode impedance is measured automatically by the EEG hardware. Electrodes with high impedance resulting from bad contact or no contact were subsequently excluded from estimation of the average activity.

#### Feature extraction

The great interest in investigations of causal relationships, particularly when dealing with neurophysiological data, has motivated the development of measures that capture such relationships. One such measure is Granger Causality (GC). GC has been developed explicitly to allow inferences about causality between two time series to be made [22]. Wiener defined causality as: “for two simultaneously measured signals, if one can predict the first signal better by incorporating the past information from the second signal than using only information from the first one, then the second signal can be called causal to the first one” [23]. Granger later gave this a mathematical formulation by using univariate and bivariate autoregressive models (AR): for two time series, **X**_1_, and **X**_2_, if **X**_1_ is influenced by **X**_2_, then the addition of past values of **X**_2_ in the regression of **X**_1_ will improve its prediction. Thus, the basic idea of GC is: for the two time series, **X**_1_ and **X**_2_, we try to predict *x*_1_(*t*+1) using (i) only past samples of **X**_1_ (univariate AR model), and (ii) past samples of both **X**_1_ and **X**_2_ (bivariate AR model). Causality can be assessed from the variances of the prediction errors of the resulting AR models.

In the univariate case,
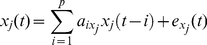
(1)where 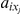 are the estimated univariate AR coefficients for the AR model of order *p*, 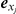 are the residuals (prediction errors) of the AR process, and *j* = 1,2.

Similarly, for the bivariate AR model:
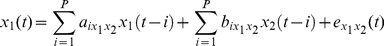
(2)where 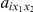, 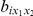 and 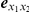 are as for the univariate AR; similarly for *x*_2_(*t*).

Let us denote the variance of the prediction errors as 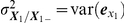 and 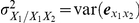 for the bivariate and univariate case respectively. Granger Causality can then be defined as:
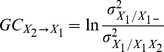
(3)If 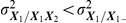 then it is implied that the prediction of **X**_1_ is improved by using past values of **X**_2_ in its prediction; thus, 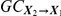 will increase. If, however, the past of **X**_2_ does not improve the prediction of **X**_1_, then 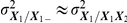, and 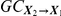 will be close to zero. Thus, by definition, 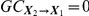 when the signals are independent, and 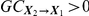 otherwise. However, in practice the latter also depends on the signals investigated and any amplitude limitations they may have. Similarly, 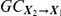 is defined accordingly. If both 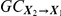 and 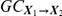 are high, then this indicates a bidirectional coupling or feedback relationship between **X**_1_ and **X**_2_. However, the existence of a strong bidirectional GC could also be due to exogenous factors, such as a common driver acting on both **X**_1_ and **X**_2_. In such a case, the remaining interdependence (instantaneous causality) between **X**_1_ and **X**_2_ is captured by the covariance of the bivariate prediction errors, based on Geweke's theorem of time series decomposition [24].

Other considerations during feature extraction included:

1. Stationarity: Granger Causality was estimated over 4-second EEG windows (window sliding by 1-s). Segments with such short duration were chosen for two reasons. Firstly, to identify cases of impending awareness as quickly as possible; and, secondly, this is common practice in EEG analysis to ensure the stationarity of the EEG segments analyzed [25]. Stationarity was specifically assessed using the Kwiatkowski–Phillips–Schmidt–Shin (KPSS) test (at a critical level of 0.01) [26], using the GCCA toolbox [27]. The segments which were found to be non-stationary were excluded from further analysis.
2. AR Modeling: The Durbin-Watson test was used to assess the residual variance [28] (function ‘*dwtest*’ in Matlab®). Any segments with autocorrelated residuals were excluded from the analysis. We also performed a consistency check [29] on the estimated AR models and excluded segments for which the fitted AR models had a consistency less than 70%. The AR model order was estimated for each patient using the Bayesian Information Criterion (BIC), which is more appropriate for neural applications [30]. However, from additional investigations, we found that utilizing a fixed AR model order of 6 for all patients did not degrade performance, as measured through the consistency of the fitted AR model. Therefore, a 6^th^ order AR model was used for all patients.
3. Artifacts: The main sources of artifacts during anesthesia are artifacts during tracheal intubation at anesthetic induction, and diathermy noise during surgery. We removed intubation artifacts by excluding the first 5 minutes following anesthetic induction; this also served the purpose of ensuring that the patient was fully unconscious. Segments which were contaminated with diathermy were excluded from further analysis. We also investigated the application of a 50-Hz notch filter for removing line noise (using the Matlab® function ‘*iirnotch*’); details can be found in the Discussion. No other artifact removal was performed.

To estimate the GC values for each 4-s EEG segment, **X**_1_ and **X**_2_ corresponded to one pair from the five predefined brain areas. Thus, applying GC on the predefined 5 brain areas resulted in 10 pairs of bidirectional GC estimates:
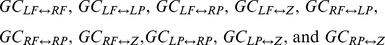
where LF: left frontal, RF: right frontal, LP: left posterior, RP: right posterior, and Z: midline. Prior to utilizing the estimated GC as a feature, the statistical significance of the observed patterns was verified using the method of phase randomized surrogate data [31]. In brief, a number of surrogate data is generated by Fourier transforming the original data, substituting the phases with random ones, and transforming back to the time domain via an inverse Fourier transform. This results in data that have the same second order properties as the measured data, but which are otherwise random.

Based on our preliminary investigations, the most characteristic change of the GC index was the significant increase of GC from frontal to posterior regions when the subject was anesthetized [19], [20]. The GC patterns from these areas were, thus, chosen as features. This resulted in feature vectors that consisted of the following 4-dimensional values:
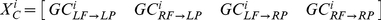
(4)where 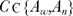 corresponds to one of the two classes, and *i* = 1,…,*N_C_* denotes the *i* ^th^ 4-s segment from all the available segments of each class (*N_C_*). Since no additional pre-processing or artifact removal was performed, a moving average filter (*n* = 10 samples) was applied on the estimated GC values to smooth out any outlier effects from the presence of artifacts.

#### Volume conduction

An important consideration in EEG analysis is volume conduction. Even though the Laplacian transform offers a solution to this problem, this acts as a bandpass spatial filter, which ‘may remove genuine source activity associated with very low spatial frequencies’ [32]. Given the high sensitivity of GC measures to any filtering [21], the use of a Laplacian transform should be avoided. In order to ensure that our findings were not simply a confound of volume conduction, we have used surrogate data to control for such artifacts. This method was proposed by Shahbazi *et al.*, and the idea is to construct surrogate data that are a superposition of independent sources that are statistically as close as possible to the original data [33]. In summary, the main steps of the method are: (1) decomposition of the original data into independent sources using Independent Component Analysis (here SOBI was used [34]); (2) shifting each *n*-th estimated source by (*n*−1)*T samples, where *T* is substantially larger than any autocorrelation time (here we found *T* = 100 adequate); and (3) constructing the surrogate data by mixing the shifted sources using the estimated mixing matrix. The connectivity measure of interest is estimated for both the original and surrogate data. If a specified effect is observed in both data sets, then the observed effect is considered as insufficient evidence for a true brain interaction. For true directional interactions GC is attenuated in the surrogate data compared to the original data, but not removed. According to Shahbazi *et al.*, the effect of volume conduction can be assessed from the regression line, *y* = *x*, where *y*: GC of surrogate data, *x*: GC for original data. If this line describes the data well, then we cannot exclude that volume conduction could be responsible for the observed effect. We assessed the goodness-of-fit of this regression line via the coefficient of determination, *r* ^2^:
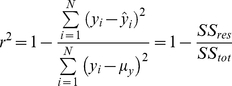
(5)where *SS_tot_* is the total sum of squares, *SS_res_* is the residual sum of squares, *μ_y_* is the mean of the observed data *y*, and 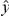 is the prediction from the linear regression *y* = *x*. From (5) it can be seen that if the model does not provide a good fit for the data, then *SS_res_*>*SS_tot_*, and *r* ^2^<0.

### Performance estimation

Classification performance was obtained for each subject over B = 200 bootstrap repetitions (sampling with replacement). For each patient the number of samples (windows) available for the ‘awake’ and ‘anesthetized’ classes were *N_aw_* and *N_an_* respectively. The size of the training set was determined as *N_train_* = min{0.8*N_aw_*,0.8*N_an_*} with maximum *N_train_* = 100. Thus, the training set was composed by randomly choosing *N_train_* windows from each class, while the remaining *N_aw_* –*N_train_* and *N_an_*–*N_train_* windows composed the test sets for class ‘awake’ and ‘anesthetized’ respectively. The number of windows available for the ‘anesthetized’ class were set to 300 for all patients (this was possible as data were available throughout the entire surgical duration for each patient), while the number of windows for the ‘awake’ class differed from patient to patient. The mean number of available windows ± standard deviation were: (i) LOC: *N_aw_* = 249.7±295.4, and *N_train_* = 77.0±28.6; and (ii) ROC: *N_aw_* = 142.3±114.1, and *N_train_* = 76.5±31.4. Classification was performed with simple Linear Discriminant Analysis (LDA), and a more complex Support Vector Machine (SVM) [35]. For the SVM classifier both linear and non-linear (Radial Basis Function with radius 1) kernels were investigated (denoted as SVM_L_ and SVM_NL_ respectively). Performance was assessed as the specificity (6), sensitivity (7) and average accuracy (8):
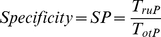
(6)

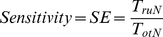
(7)

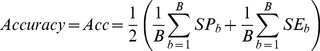
(8)where *T_otP_*(*T_otN_*) is the total number of ‘ground truth’ positive (negative) examples, *T_ruP_* is the number of ‘ground truth’ positive examples correctly classified as positive, and *T_ruN_* is the number of ‘ground truth’ negative examples correctly classified as negative. In the following investigations, examples of class ‘awake’ were considered as positive, while examples from the ‘anesthetized’ class were considered as negative.

An additional consideration is that a patient awaking from surgery does not regain full alertness until some time afterwards; this time frame is very much dependent on the rate at which each person is able to metabolize the administered drugs. This implies that the awareness state of a patient at ROC could be more similar to the awareness state of the patient in the case that awareness is experienced during surgery. In order to investigate whether wakefulness prior to anesthetic administration and wakefulness at the end of surgery differed, we performed two separate investigations utilizing data extracted around (a) the marker for anesthetic administration; and (b) the marker for recovery of consciousness. Therefore, we performed separate classifications with different classifiers for these two cases.

## Results

**Figure 1:**
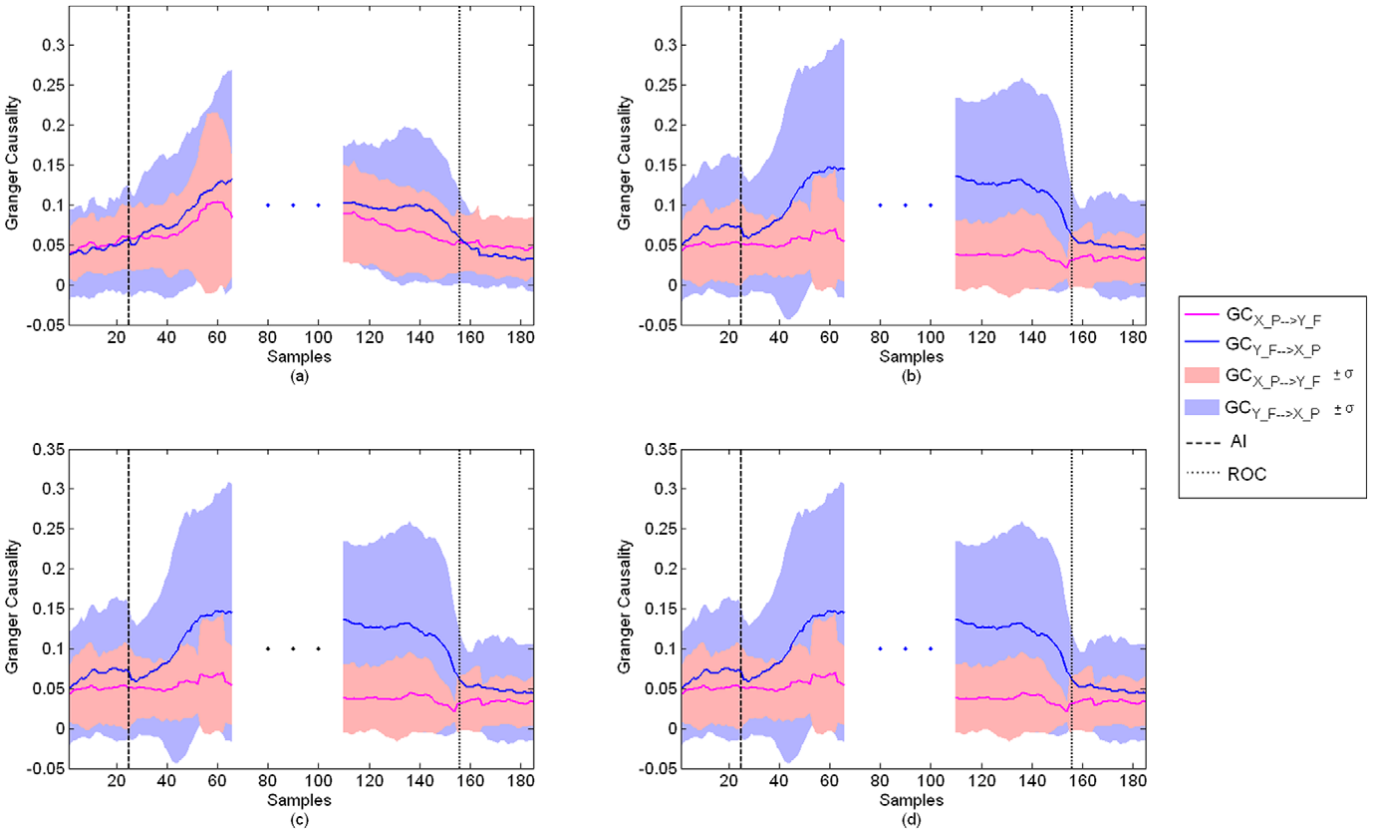
Average fronto-posterior Granger Causality patterns. Maroon line: posterior→frontal direction. Blue line: frontal→posterior direction. GC between (a) left frontal – left posterior, (b) right frontal – left posterior, (c) left frontal – right posterior; (d) right frontal – right posterior. Shaded areas: mean GC ± standard deviation. An increase in fronto→posterior GC after anesthesia induction is observed. Vertical lines indicate anesthetic induction (AI) and recovery of consciousness (ROC). As expected, the fronto→posterior GC returns to baseline at recovery of consciousness. Subjects with no GC over the right posterior area due to bad electrode contact were excluded from the average (2 patients). X-axis in arbitrary samples.

**Figure 2:**
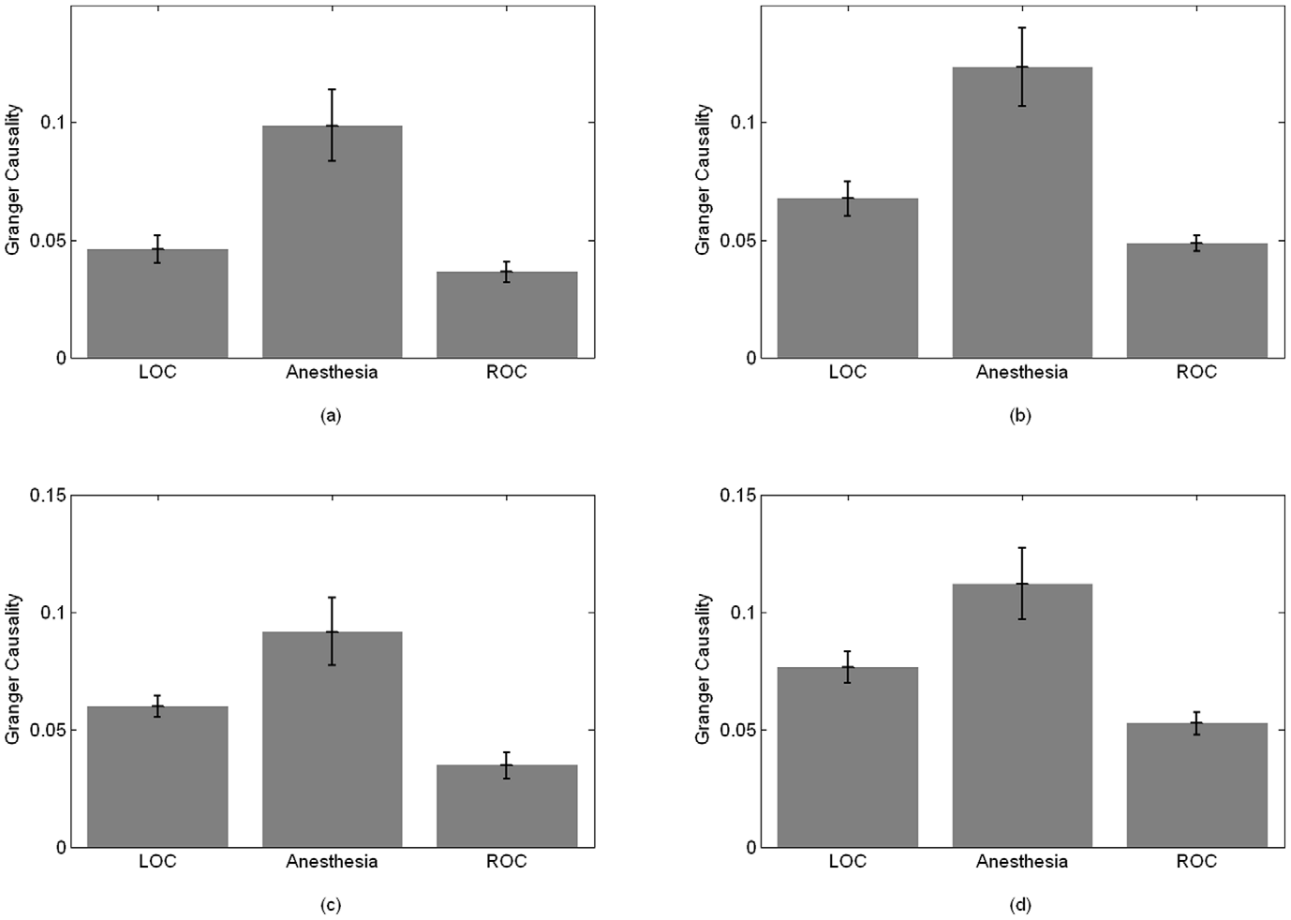
Patient-wise average GC values ± standard deviation (error bars). 50-second segments of ‘Awake’ (pre-LOC, post-ROC) and ‘Anesthetized’ (mean GC for post-LOC and pre-ROC) states. (a) GC_LF→LP_, (b) GC_RF→LP_, (c) GC_LF→RP_, and (d) GC_RF→RP_. The differences in GC between ‘Awake’ and ‘Anesthetized’ states are statistically significant (ANOVA F-test, α = 0.05, p = 0).

**Figure 3:**
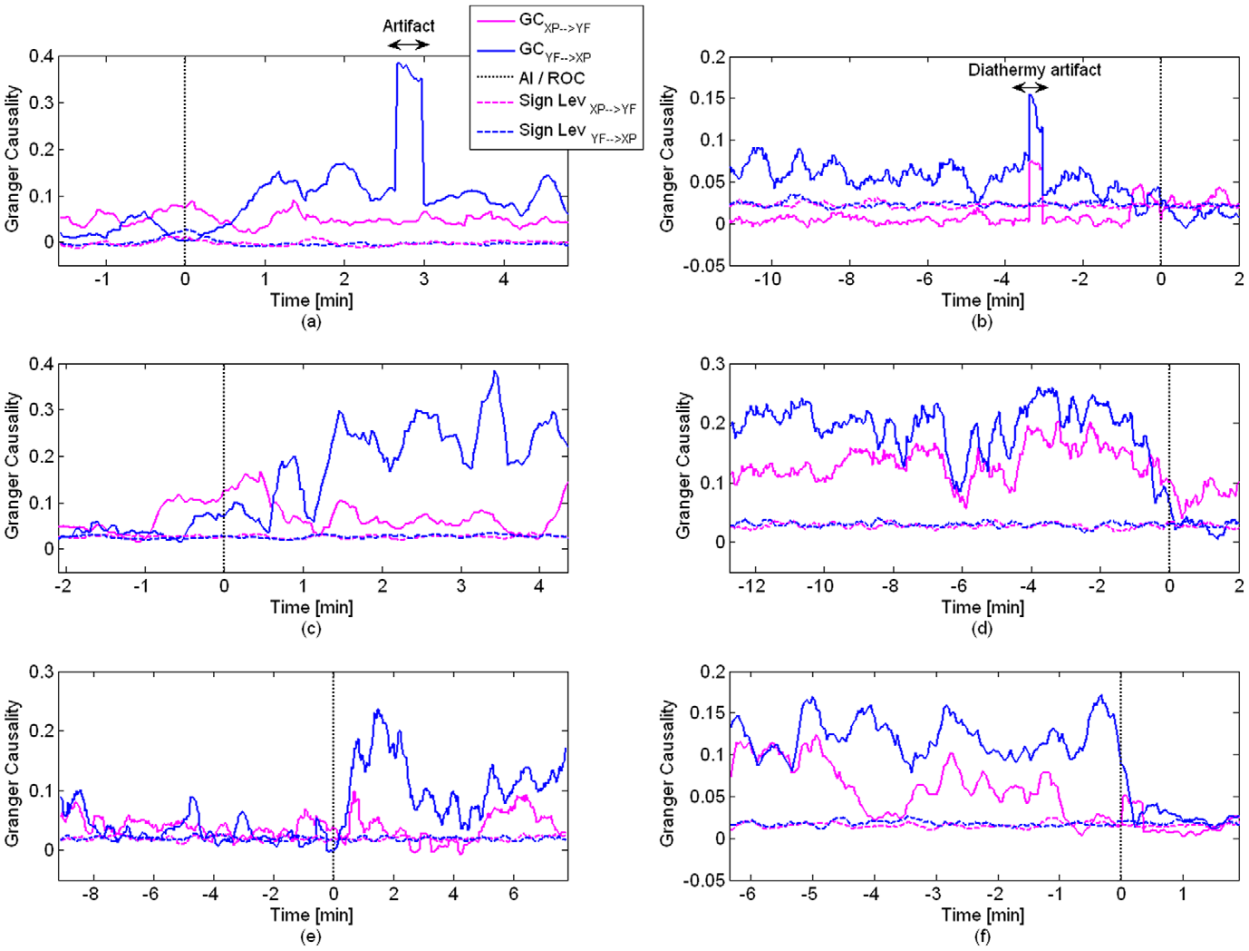
Individual GC values for LOC and ROC conditions with 95% significance level. (a) GC_LF↔LP_ for patient S11 at LOC. (b) GC_RF↔LP_ for patient S1 at ROC. (c) GC_LF↔RP_ for patient S13 at LOC. (d) GC_RF↔RP_ for patient S17 at ROC. (e) GC_RF↔LP_ for patient S8 at LOC. (f) GC_LF↔RP_ for patient S21 at ROC. Vertical line indicates anesthetic administration ((a), (c), (e)), and recovery of consciousness ((b), (d), (f)). Outlier GC values due to the presence of artifacts in the raw EEG signal are also visible in (a) and (b).

Figure 1 shows the average fronto-posterior GC values at LOC and ROC (a moving average filter of length 10 samples was applied to the GC values). The increase of GC from frontal to posterior areas with LOC and its reversal at ROC can be clearly seen. Even though the actual GC values display large inter-subject variability, similar patterns are observed for all patients studied. These changes in the GC observed while the patient is unconscious (post-LOC and pre-ROC GC values) are statistically different from the baseline values observed while the subject is awake (pre-LOC and post-ROC) (ANOVA F-test, α = 0.05, p = 0). Figure 2 shows the subject-wise average GC values for the two states ‘Awake’ and ‘Anesthetized’ estimated for 50-second segments of pre-LOC, ‘anesthesia’ and post-ROC. In addition, the fronto-posterior increase in GC values was found to be statistically significant. This can be seen in figure 3, which shows representative examples of fronto-posterior GC for individual patients. Statistical significance was assessed using the method of phase randomized surrogate data and the significance level was estimated as the maximum GC obtained from the surrogate datasets at each EEG segment. Testing the estimated GC for artifacts of volume conduction resulted in negative coefficients of determination (*r* ^2^), hence we can deduce that the observed GC cannot be fully explained as an effect of volume conduction. We can also rule out that the observed effects are a result of the use of neuromuscular blockers, as not all patients received neuromuscular blockers for the entire surgical duration (see tables 1 and 2). This is common when the surgical duration is relatively short.

**Table 1:** Average classification performance for each subject at anesthesia induction (LOC).

	Specificity			Sensitivity			Accuracy		
Subject	SVM_NL_	SVM_L_	LDA	SVM_NL_	SVM_L_	LDA	SVM_NL_	SVM_L_	LDA
**S1**	1.000	0.999	1.000	0.989	0.992	0.980	0.995	0.996	0.990
**S2**	0.948	0.890	0.849	0.979	0.923	0.951	0.964	0.907	0.900
**S3**	0.902	0.947	0.962	0.928	0.835	0.827	0.915	0.891	0.896
**S4**	1.000	1.000	1.000	0.999	0.999	0.983	1.000	1.000	0.992
**S5**	1.000	1.000	1.000	1.000	1.000	0.997	1.000	1.000	0.998
**S6**	0.891	0.884	0.868	0.953	0.925	0.930	0.922	0.905	0.899
**S7**	1.000	1.000	1.000	1.000	1.000	1.000	1.000	1.000	1.000
**S8**	0.968	0.949	0.953	0.985	0.977	0.973	0.977	0.963	0.963
**S9**	1.000	1.000	1.000	0.992	0.971	0.864	0.996	0.986	0.932
**S10**	1.000	1.000	1.000	0.986	0.980	0.977	0.993	0.990	0.989
**S11** 1	1.000	1.000	1.000	0.999	1.000	0.966	0.999	1.000	0.983
**S12** 2	0.997	0.835	0.845	0.975	0.774	0.764	0.986	0.805	0.805
**S13** 1	1.000	1.000	1.000	0.978	0.980	0.970	0.989	0.990	0.985
**S14**	0.968	0.974	0.973	0.985	0.975	0.990	0.977	0.975	0.981
**S15** 1,2	0.997	0.996	0.985	0.981	0.981	0.965	0.992	0.989	0.975
**S16** 1	1.000	1.000	1.000	1.000	1.000	0.988	1.000	1.000	0.994
**S17**	0.925	0.925	0.838	0.978	0.978	0.989	0.951	0.951	0.913
**S18** 1	1.000	1.000	1.000	1.000	1.000	1.000	1.000	1.000	1.000
**S19** 1	1.000	1.000	1.000	1.000	1.000	0.957	1.000	1.000	0.979
**S20**	0.980	0.940	0.940	0.987	0.962	0.941	0.984	0.951	0.941
**S21** 1	0.976	0.978	0.988	0.968	0.962	0.933	0.972	0.970	0.960
**TOTAL**	**0.979**	**0.968**	**0.962**	**0.984**	**0.963**	**0.950**	**0.981**	**0.965**	**0.956**

1 : Patient administered a very small quantity of neuromuscular blocking agent (<4 mg) at induction only to facilitate tracheal intubation.

2 : Maintenance with sevoflurane.

Performance estimated with nonlinear and linear Support Vector Machine (SVM_NL_ and SVM_L_ respectively), and Linear Discriminant Analysis (LDA). ‘TOTAL’ indicates the average performance over all patients.

**Table 2:** Average classification performance for each subject at recovery of consciousness (ROC).

	Specificity			Sensitivity			Accuracy		
Subject	SVM_NL_	SVM_L_	LDA	SVM_NL_	SVM_L_	LDA	SVM_NL_	SVM_L_	LDA
**S1**	0.996	0.990	0.945	0.920	0.902	0.771	0.958	0.946	0.858
**S2**	0.878	0.794	0.817	0.869	0.762	0.743	0.873	0.778	0.780
**S3**	0.943	0.930	0.931	0.989	0.987	1.000	0.966	0.959	0.965
**S4**	0.917	0.842	0.845	0.899	0.721	0.715	0.908	0.782	0.780
**S5**	0.797	0.757	0.750	0.853	0.918	0.944	0.825	0.838	0.847
**S6**	0.761	0.701	0.704	0.734	0.563	0.563	0.748	0.632	0.633
**S7**	0.988	0.989	1.000	0.993	0.992	0.977	0.990	0.990	0.988
**S8**	0.950	0.797	0.823	0.846	0.768	0.758	0.898	0.782	0.791
**S9**	1.000	1.000	1.000	1.000	1.000	0.978	1.000	1.000	0.989
**S10**	0.999	0.991	1.000	0.909	0.911	0.786	0.954	0.951	0.893
**S11** 1	1.000	1.000	1.000	0.995	0.995	0.992	0.998	0.997	0.996
**S12** 2	1.000	1.000	1.000	0.962	0.959	0.872	0.981	0.980	0.936
**S13** 1	1.000	0.998	1.000	0.976	0.975	0.902	0.988	0.987	0.951
**S14**	0.939	0.925	0.949	0.928	0.902	0.910	0.934	0.913	0.929
**S15** 1 **^,^** 2	1.000	1.000	1.000	1.000	1.000	0.989	1.000	1.000	0.984
**S16** 1	1.000	1.000	1.000	0.948	0.948	0.962	0.974	0.974	0.981
**S17**	0.974	0.973	0.999	0.923	0.896	0.877	0.949	0.935	0.938
**S18** 1	1.000	1.000	1.000	0.994	0.988	0.981	0.997	0.994	0.991
**S19** 1	0.987	0.998	1.000	0.975	0.953	0.977	0.981	0.976	0.988
**S20**	0.931	0.874	0.853	0.948	0.887	0.906	0.940	0.880	0.879
**S21** 1	0.997	0.998	1.000	0.998	0.998	0.997	0.997	0.998	0.998
**TOTAL**	**0.955**	**0.931**	**0.934**	**0.940**	**0.906**	**0.885**	**0.946**	**0.919**	**0.909**

1 : Patient administered a very small quantity of neuromuscular blocking agent (<4 mg) at induction only to facilitate tracheal intubation.

2 : Maintenance with sevoflurane.

Performance estimated with nonlinear and linear Support Vector Machine (SVM_NL_ and SVM_L_ respectively), and Linear Discriminant Analysis (LDA). ‘TOTAL’ indicates the average performance over all patients.

**Figure 4:**
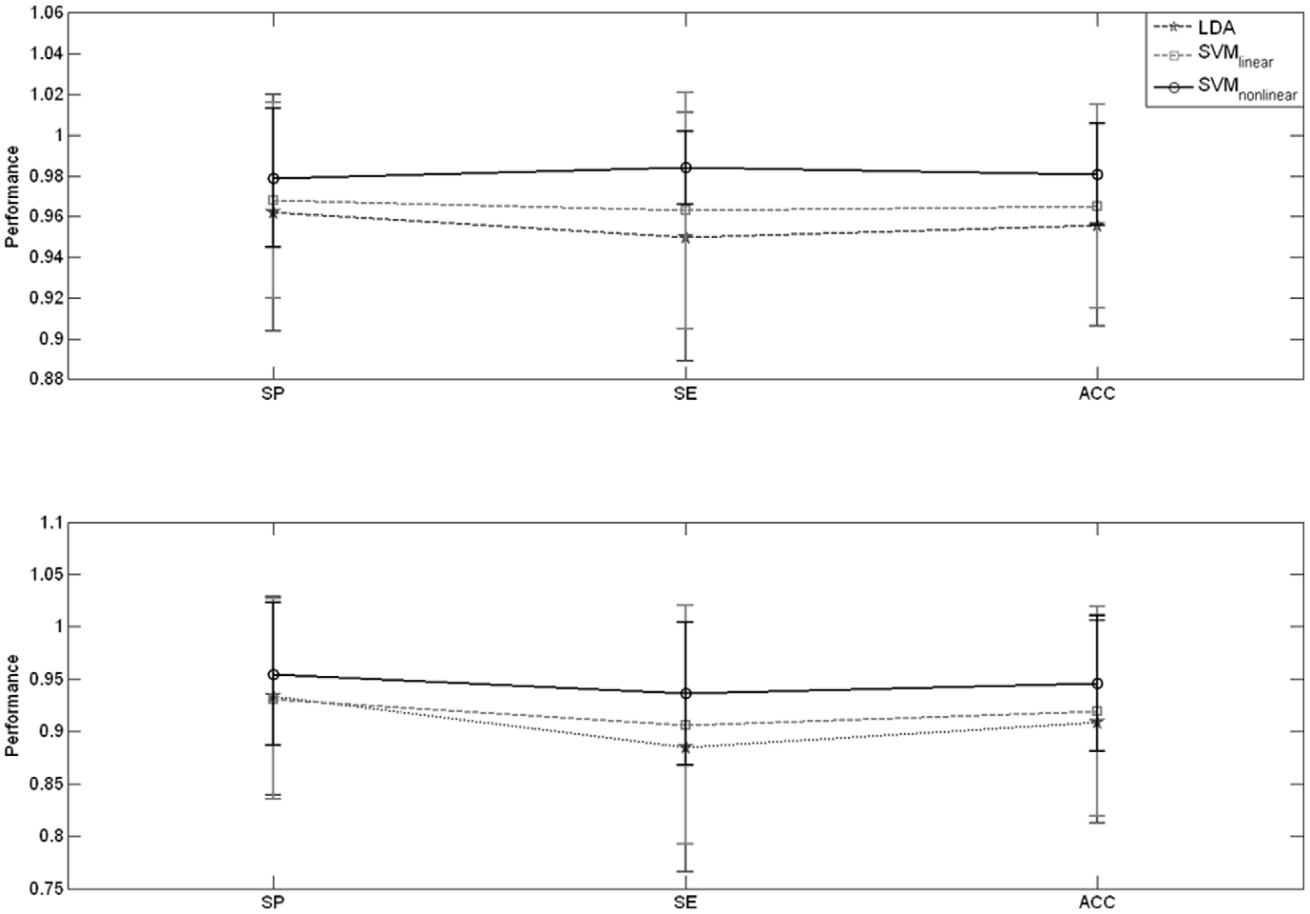
Average classification performance (mean ± standard deviation) for LOC (top) and ROC (bottom) conditions.

Tables 1 and 2 show the average Specificity (SP), Sensitivity (SE), and Accuracy (Acc) for each subject, as well as total SP, SE and Acc averaged over all subjects, for data from LOC and ROC respectively. Classification for LDA, SVM_L_ and SVM_NL_ is displayed on the same table. The average classification performance over all subjects can also be seen in figure 4, together with error bars (standard deviation). The best average performance obtained is (1) LOC condition: SVM_NL_ with (mean ± standard deviation) SP 0.98±0.034, SE 0.98±0.018 and Acc 0.98±0.025; and (2) ROC condition: SVM_NL_ with (mean ± standard deviation) SP 0.94±0.068, SE 0.96±0.068 and Acc 0.95±0.065. The nonlinear SVM displays better performance, with differences in performance being statistically significant only when compared to LDA (see table 3 for details; statistical significance assessed with one-way ANOVA F-test, α = 0.05). Statistical differences in performance between LOC and ROC conditions are shown in table 4 (statistical significance assessed with one-way ANOVA F-test, α = 0.05). The goodness-of-fit of the AR models was assessed via the model consistency, which shows how much of the data variance is captured by the model (100% indicates an ideal model). The patient-wise average consistency for all segments used in the analysis is 98.2±0.955% (mean ± standard deviation). The KPSS test confirmed the appropriateness of using 4-s segments, as the total number of segments that were excluded from analysis due to non-stationarity was 2.6% (LOC) and 3.8% (ROC).

**Table 3:** Statistical significance of linear Vs non-linear classification.

Perf.	LOC			ROC		
SP		SVM_L_	LDA		SVM_L_	LDA
	**SVM_NL_**	F = 0.75, p = 0.39	F = 1.28, p = 0.26	**SVM_NL_**	F = 0.85, p = 0.36	F = 0.68, p = 0.41
	**SVM_L_**		F = 0.11, p = 0.74	**SVM_L_**		F = 0.01, p = 0.93

SE		SVM_L_	LDA		SVM_L_	LDA
	**SVM_NL_**	F = 2.5, p = 0.12	F = 6.02, p = 0.02^*^	**SVM_NL_**	F = 1.1, p = 0.30	F = 2.98, p = 0.09
	**SVM_L_**		F = 0.5, p = 0.48	**SVM_L_**		F = 0.35, p = 0.56

Acc		SVM_L_	LDA		SVM_L_	LDA
	**SVM_NL_**	F = 1.75, p = 0.19	F = 4.39, p = 0.04^*^	**SVM_NL_**	F = 1.06, p = 0.31	F = 2.01, p = 0.16
	**SVM_L_**		F = 0.36, p = 0.55	**SVM_L_**		F = 0.09, p = 0.76

Statistical significance of differences in performance of the different classifiers at loss and recovery of consciousness (LOC and ROC respectively). Classifiers: linear (SVM_L_) and nonlinear (SVM_NL_) Support Vector Machine, and Linear Discriminant Analysis (LDA). Performance (Perf.) estimated as specificity (SP), sensitivity (SE), and accuracy (Acc). Significance was estimated with one-way ANOVA F-test (α = 0.05; F_crit_(1,41) = 4.079), and significant differences are marked with *.

**Table 4:** Statistical significance of LOC Vs ROC classification.

Perf.	SVM_L_	SVM_NL_	LDA
**Specificity**	F = 2.37, p = 0.13	F = 2.01, p = 0.16	F = 1.32, p = 0.26
**Sensitivity**	F = 4.19, p = 0.05^*^	F = 9.74, p = 0.003^*^	F = 5.04, p = 0.03^*^
**Accuracy**	F = 3.6, p = 0.06	F = 5.55, p = 0.02^*^	F = 3.81, p = 0.06

Statistical significance of differences between loss and recovery of consciousness conditions (LOC and ROC respectively). Classifiers: linear (SVM_L_) and nonlinear (SVM_NL_) Support Vector Machine, and Linear Discriminant Analysis (LDA). Significance was estimated with one-way ANOVA F-test (α = 0.05; F_crit_(1,41) = 4.079), and significant differences are marked with *.

Table 5 provides a quantitative comparison with other work [36], [37], [38], [39], [40], [41], [42], [43], [44], [45]. Performance is reported as ‘accuracy’ (correct classification of segments corresponding to LOC and ROC) or ‘prediction probability’ (correlation with concentration of anesthetic agent or observed anesthetic depth as scored by experts). From table 5 it can be seen that the performance achieved with GC features is considerably better than other techniques and devices that are commercially distributed.

**Table 5:** Quantitative comparison with other methods reported in the literature.

Ref.	Accuracy	Prediction Probability	Features
*This work*	0.98		Granger Causality
[44]		0.92	Narcotrend™ monitor
[36]	0.86		Recurrence quantification analysis
[37]		0.86	Approximate Entropy
		0.86	Spectral edge frequency
		0.78	Median frequency
		0.82	BIS® monitor
[41]		0.77	Approximate Entropy
		0.87	Permutation Entropy
		0.87	Order Recurrence Rate
		0.87	Phase coupling of order patterns
[42]	0.85		Approximate Entropy
[40]		0.86	Permutation Entropy
		0.79	Approximate Entropy
[38]		0.84	Hilbert-Huang state entropy
[45]	0.69		Time Encoded Signal Processing and Recognition (TESPAR)
[43]		0.87	BIS® monitor
		0.89	Datex-Ohmeda S/5 Monitor (State Entropy)
		0.88	Datex-Ohmeda S/5 Monitor (Response Entropy)
[39]	0.93		Complexity based on Lempel-Ziv
	0.89		Approximate Entropy
	0.76		Spectral Entropy
	0.64		Median Frequency

## Discussion

The ability to discriminate between ‘Awake’ and ‘Anesthetized’ state is important for depth of anesthesia monitors. Using GC as features, we were able to obtain high sensitivity, specificity and accuracy. Despite the inter-subject variability in the actual GC values for each subject, the GC patterns displayed the same trend for all subjects. Even though SVM is a powerful classifier suitable for complex high-dimensional problems, it was chosen here specifically for this simpler low-dimensional problem, as it allows us to study both linear and non-linear classification utilizing a single technique. Even though linear classification was outperformed by non-linear classification, the differences in performance are not statistically significant. This implies that the GC features utilized are linearly separable and, from a statistical perspective, it is not necessary to introduce a more complex non-linear classifier with increased computational cost. Therefore, a much simpler linear classifier, such as LDA, or even a technique based on some form of adaptive threshold estimation, could be utilized. The latter could also be more appropriate for real-time applications and remains the subject of future investigations.

Pairwise time-domain GC analysis has received some criticism, mainly regarding the interpretation of the resulting causality relationships. A main limitation is that one cannot distinguish between direct and indirect causal relationships when performing pairwise GC analysis. This is related to the issue of spurious causality that can appear between two processes when both are influenced by external sources that are not taken into account [46]. In cases when the interdependence between the two time series cannot be fully explained by their interactions, one can examine the covariance of the noise terms in the estimated AR models, which captures the remaining interdependence [30]. Another solution is provided by conditional GC, which conditions the estimated GC onto external sources [47]. In order to infer a more precise structural causality, in theory one must include *all* sources of influence into the estimation. However, in practice this is always unfeasible and, as a result, conditional methods will always be provisional [48]. In a recent study by Wang *et al.*, it was shown that both pairwise and blockwise approaches to GC estimation gave consistent results [49]. Pairwise time-domain GC is a valid methodology with a lot to offer in terms of inferring causality patterns, as long as the limitations mentioned above are taken into consideration (for some examples of recently published articles utilizing pairwise GC analysis see [50], [51], [52]).

The difference between classification performance for examples from LOC and ROC indicates that both conditions show some statistically significant variations (table 4). This could be an indication of differences between brain activity during wakefulness before and after administration of the anesthetics.

The marker for ROC indicates that the subject has regained consciousness. The administration of anesthetics had been switched off a few minutes prior to this event. Should the estimated GC features have been a reflection of the metabolic decrease of the anesthetic agent, the decrease in the values of GC would be gradual and not sharp as observed. Thus, there would not have been a clear boundary between the GC features for each class, leading to lower classification accuracy. However, the high performance is an indicator that GC features reflect the points at which consciousness is lost and recovered. This provides strong support for the use of such features in a DOA monitor as a change in the patient's state of awareness would be promptly captured. It is also possible that some of the segments used in the analysis may contain data from the start/end of the surgical procedure, and it is known that surgical noxious stimuli, e.g., tends to lighten the level of hypnosis [53]. However, the observed GC patterns remain stable from the onset of LOC to ROC and are neither affected by, nor are a direct result of, the surgery itself. In addition, despite the large inter-subject variability in the actual GC values, the observed GC patterns remain robust between subjects and different anesthetic regimes. This strengthens the belief that GC is related to the general physiological mechanism underlying anesthetic administration. This is in contrast to current DOA monitors, which use EEG activity as a proxy for consciousness, and which do not take into account the inter-subject variability.

Bidirectional interaction or strong unidirectional interaction in the presence of a common input as captured by GC are related to mechanisms of information flow in cortical circuits, in terms of the anatomical connectivity principle of reciprocity in the cortex or the collective activation of cortical regions projecting to the measured sites respectively [54]. Therefore, the observed GC patterns are particularly interesting in terms of a physiological interpretation of the disruption of consciousness by anesthetics. It is now believed that anesthetics do not block incoming sensory information, but interfere with the coherent interpretation of it by the brain such that it is not consciously perceived [1], [3], [4], [55], [56]. Evidence from various connectivity measures suggests that the effective connectivity between lateral antero-posterior networks is an important mechanism for this information integration and, thus, for conscious perception itself [5], [57], [58], [59], [60]. Both this disconnection, as well as the hypersynchronisation of neuronal activity seen during deeper anaesthesia, leads to loss of the brain's integrative capacities [61]. A part of this neurophysiological mechanism is also common for other unconsciousness-related states, such as deep sleep and vegetative state [62], [63]. Similar findings were reported in a study of deep sleep by Massimini *et al.*, where it was shown that the slow oscillations observed during deep sleep are travelling waves that sweep the cortex in an antero-posterior direction [64]. It is possible that the slow waves that characterize anaesthesia are also travelling waves with a similar underlying physiological mechanism. When interpreting the observed GC patterns one must remember that GC is based on a statistical concept. Hence, causality captured by GC could be mediated either by direct or indirect pathways through the cortex or subcortical structures and does not in itself provide proof of a direct activation from one neuronal structure to another via an axonal pathway. Therefore, the increase in GC from frontal to posterior regions does not necessarily imply that this is mediated through a direct connection between the two regions. The observed changes in the GC reflect the disruption of information flow in terms of effective connectivity, as captured non-invasively through the EEG. In addition, even though the approach of estimating GC between regional time series is followed in many studies, it is understood that the process of averaging the spontaneous EEG over multiple sensors results in loss of some information. Hence, it is likely that the GC estimated between such regional averages may present some differences compared to pair-wise or block-wise GC estimations [65]. One should bear this in mind when the aim is the precise investigation of effective connectivity. However, here we are interested in the broad characteristic changes in the observed GC patterns related to anesthetic administration, which can be used reliably for monitoring awareness during surgery.

Regarding the choice of an appropriate order for the AR model utilized, this is non-trivial: if the order is too low the properties of the signals are not captured, however if the order is too high then any measurement noise or inaccuracies are also represented and the resulting model is not a reliable representation of the signal [66]. This is particularly true for EEG signals, where increasing the model order reduces the value of the order estimation criteria asymptotically without observing a true minimum. Here, the optimum AR order was estimated using the BIC.

**Figure 5:**
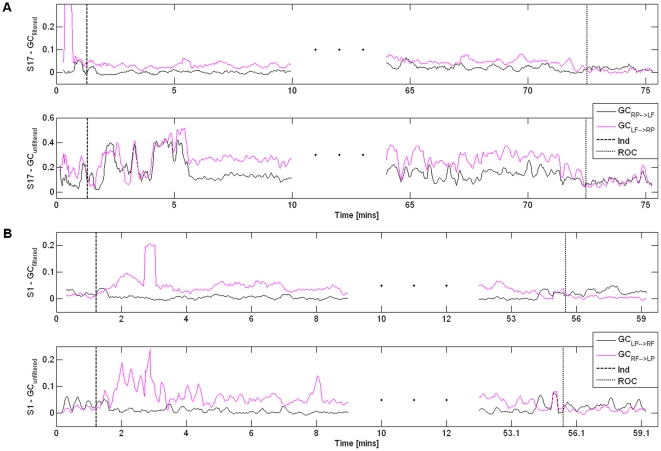
Granger Causality features for 50-Hz notch filtered and unfiltered data. A: GC between left frontal and right posterior areas for patient S17; B: GC between right frontal and left posterior areas for patient S1. GC is shown for notch filtered EEG (top panels of A and B) and unfiltered EEG (bottom panels of A and B). Vertical lines denote anesthetic induction (dashed line) and recovery of consciousness (dotted line). The effects of diathermy artifacts on the GC estimates can also be identified as sharp outliers.

Another important consideration is the presence of 50-Hz line noise, which can be removed easily using a 50-Hz notch filter. The use of filtering in GC analysis has raised some contradictive opinions (see work by Florin *et al.* [21], Seth [27], and Barnett & Seth [67]). The general agreement between the different works is that despite the theoretical invariance of GC to linear operations, in practice filtering has an effect on GC estimations: it can induce time-domain causal network artifacts, and has a substantial impact on statistical significance testing [67]. The effects on GC estimation are a consequence of increased empirical AR model order from filtering. The main motivation for using a notch filter is to remove the non-stationarity induced by line noise. However, ‘*although strictly speaking the process with an added sinusoid is non-stationary, in finite sample it may be approximated and modeled*’ as a vector autoregressive process of order *p* [67]. Here, we have found that the application of a 50-Hz notch filter did not remove any non-stationarities present. In the majority of cases notch filtering did not affect the underlying GC patterns, however it reduced the differences in the fronto-posterior GC values (see figure 5), which resulted in a less effective discrimination between wakefulness and anesthesia.

An ideal DOA monitor should display 100% SE and SP. However, it is very difficult to have an ideal monitor and in the majority of cases a compromise between SE and SP must be made. But what does this compromise translate to in terms of a DOA monitor? Let us first consider what SE and SP imply for a DOA monitor. Ideal SP means that all events of awareness are captured by the monitor. This implies that an alarm is raised and, in such a case, appropriate actions, such as administration of an anesthetic bolus, would have to be taken by the anesthetist to ensure adequate anaesthesia. Ideal SE would imply that when the patient is adequately anesthetized, the DOA monitor reflects this and no further action is needed. Now let us consider the consequences of non-ideal SE and SP. In case of low SE, false alarms would be raised by the monitor, falsely indicating that the patient is awake. If the anesthetist takes action in such a case, the consequences could be disastrous. In case of low SP, the monitor would fail to raise the alarm in some cases of awareness. The anesthetist would take no action and the patient would continue being aware, with possible psychological consequences to the patient. It can be seen that in the case of a DOA monitor, both SE and SP are equally as important and no sacrifice of one should be made for the other. Using GC as a feature, even though SE and SP are not ideal, both are at a similarly high level. Thus, neither is sacrificed for the other.

The feasibility of utilizing Granger Causality, a measure quantifying linear bidirectional signal interactions, as a feature for discriminating between brain activity from awake and anesthetized subjects has been investigated. Our findings support the use of GC estimated in the direction of anterior to posterior brain areas as a feature to discriminate between the EEG of an awake and anesthetized subject. High sensitivity, specificity and average accuracy were obtained for both linear and non-linear classification. The findings suggest that GC-based features are linear, thus the use of a complex non-linear classifier is not necessary. Thus, it may even be possible to employ some form of a more sophisticated and adaptive threshold for classification purposes, which would perhaps be more appropriate for the future development of a DOA monitor. The threshold would need to be adaptive as, despite the same GC patterns observed in all subjects, there is large inter-subject variability in the actual GC values that characterize ‘Awake’ and ‘Anesthetized’ states in each patient. A monitor based on adaptive classification would be advantageous over current DOA monitors, whereby the range discriminating the two states is fixed. Future work will focus on identifying the location of a small number of electrodes that can be utilized successfully in a DOA monitor, instead of utilizing the average activity of all available electrodes.
